# The Impact of Diet on Microbiota Evolution and Human Health. Is Diet an Adequate Tool for Microbiota Modulation?

**DOI:** 10.3390/nu12061654

**Published:** 2020-06-02

**Authors:** Laura Moles, David Otaegui

**Affiliations:** Multiple Sclerosis Group, Neurosciences Area, Biodonostia Health Research Institute, C. P. 20014 Donostia-San Sebastián, Spain; david.otaegui@biodonostia.org

**Keywords:** gut microbiota, Western diet, chronic disease, prebiotic, probiotic

## Abstract

The human microbiome is emerging as an interesting field in research into the prevention of health problems and recovery from illness in humans. The complex ecosystem formed by the microbiota is continuously interacting with its host and the environment. Diet could be assumed to be one of the most prominent factors influencing the microbiota composition. Nevertheless, and in spite of numerous strategies proposed to modulate the human microbiota through dietary means, guidelines to achieve this goal have yet to be established. This review assesses the correlation between social and dietary changes over the course of human evolution and the adaptation of the human microbiota to those changes. In addition, it discusses the main dietary strategies for modulating the microbiota and the difficulties of putting them properly into practice.

## 1. Introduction

The human gut microbiota is a complex ecosystem formed by thousands of microorganisms that play an important role in human immune and metabolic functions, among others. It is estimated that more than 1000 species and 3 × 10^13^ microbial cells live in or on us, being similar in number to human cells [1,2,3]. In terms of complexity and richness, the microbiota is even larger considering its genome (the microbiome). Specifically, the human microbiome has at least 100-fold more genes than the human genome; besides this, only 10% of the microbiome is shared between individuals. Therefore, the human microbiome is a unique fingerprint, and its richness and variability may explain its ability to adapt fast to environmental conditions [4,5,6].

**Figure 1:**
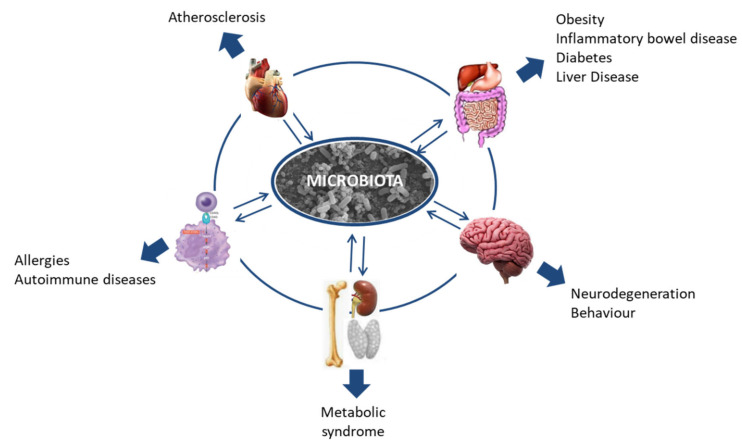
Host microbiota interactions and their relationship with disease.

The human microbiome is a relatively new field, but in recent years research into it has been increasing exponentially. The importance of the microbiota was underlined by it starting to be considered an organ in itself [4,5,7,8,9]. As the so-called “forgotten organ” [10] and considering its wide-ranging interaction with the host, research in this field could contribute to our understanding of many health problems that, so far, have proven difficult to tackle. In this context, the association of the human microbiota with health and disease is being intensively studied, and every day evidence emerges relating dysbiosis in the microbiota to more health problems, including diverse gastrointestinal and neurological disorders such as colitis, obesity, irritable bowel syndrome, Alzheimer’s disease, autism, or multiple sclerosis; allergies; and some types of cancer (Figure 1) [11,12,13,14,15,16,17].

The gut microbiota is especially moldable during infancy and notably stable in adulthood [18]. The limited microbiota present at birth undergoes dramatic changes before reaching the relative equilibrium that is characteristic of adulthood [7,8,9,19,20,21]. It is precisely in infancy when factors modulating the microbiota have the most marked influence [8,18,22]. Diet has been recognized as one of the strongest modulators of infant microbiota. In fact, numerous studies have described differences in the gut microbiota of breastfed and formula-fed infants [23,24]. It is believed that once the microbiota reaches an equilibrium (at 2–3 years of life), it is much more difficult to restore and modulate its composition. Once in adulthood, the gut microbiota remains relatively stable but diet continues to determine its composition. Studies carried out evaluating the microbiota associated with different diets in adulthood agree on the dominant presence of *Prevotella* in the gut of vegetarians, while levels of the genus *Bacteroides* and overall levels of the phylum Firmicutes are higher in people following diets high in protein and animal fats [25,26,27].

Consequently, it seems evident that our diet has the potential to modulate our microbiota. In this context, the aim of this study is to outline the challenges in dietary modulation of the microbiota, reviewing evolutionary changes in both diet and gut microbiota, their potential relationship, and consequences for human health and subsequently examining the strategies available for modulating the microbiota.

## 2. Dietary Changes across Human Evolution

Nutrition is one of the basic needs for a living being to survive and grow. Without a doubt, the human diet has dramatically changed from the time of first hominids to the present, and the changes have been especially fast and marked over the last 100 years [28]. Here, we describe the main features that have characterized these modifications in human diet.

### 2.1. Ancient Diet

The first hominids based their diet on plants gathered and animals hunted in the wild. Though relatively little is known about this time, it is believed that plants were the main foods eaten, while meat was limited to days on which hunting was successful. It is important to highlight that the ratios of plant to animal contributions of the hunter-gatherer diet are still controversial [29,30]; nevertheless, data from the current hunter-gatherer populations around the world suggest the predominance of plant food [22,31,32,33,34,35,36,37]. The development of the ability to control fire had a great impact on many aspects of human life. It provided protection from predators and warmth and light and was a determinant factor in the development of cooking. Cooking contributes to food energy accessibility by the efficient denaturing of proteins and starch gelatinization; it also preserves foods for longer periods by substantially reducing foodborne pathogens [38,39,40].

Another feature that caused marked dietary changes was the domestication of plants and animals. Agriculture allowed the availability of food to, more or less, meet the demand, and is considered a key element in the emergence of community life and civilization [38]. The spread of agriculture had other consequences, however; in particular, it led to a reduction in nutritional intake diversity.

### 2.2. First Civilization’s Diet

Populations from the first civilizations were able to produce their own food to meet their energy requirements. Dietary patterns were characterized by the development of the first fermentable foods, such as bread, beer, yoghurt, and wine. Furthermore, for centuries—though there were differences between civilizations and cultures—dietary habits were generally based on the consumption of carbohydrate-dominant foods (such as potato, rice, maize, wheat, and vegetables), probably because these were the most easily accessible [41,42].

**Figure 2:**
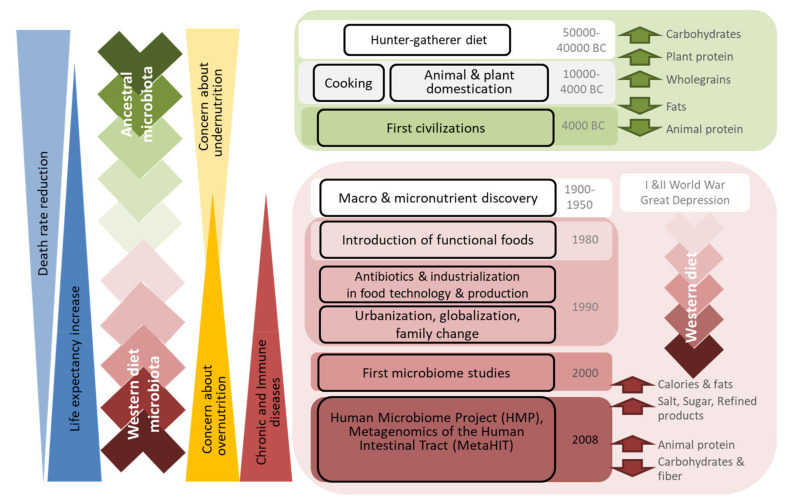
Drivers of dietary trends and their relation to microbiota composition and changes in human health.

Protein intake was primarily from legumes, as proteins of animal origin were only consumed occasionally [38,41,42,43]. Animal domestication facilitated access to meat and animal-derived products; nevertheless, cattle slaughter was commonly carried out only once or twice a year, and the meat obtained was used to supply whole families. On the other hand, the techniques used to preserve meat and fish were still quite limited; epidemics, famines, and wars marked civilizations for long periods, restricting access to valuable products, including animal-derived foods. Therefore, animal proteins remained a minor component of diets [38] (Figure 2).

### 2.3. Modern Diet

Demographic and lifestyle changes, such as urbanization, the abandonment of rural areas, and the increase in women working outside the home, have marked current populations. Through the 1990s, the worldwide growth in the use of antibiotics and industrialization in ranching and agro industries led to a new revolution in food technology and production. Together, these changes had a huge impact on food production and dietary habits [38].

On the one hand, globalization and advances in agriculture have nearly eliminated the seasonality of foods in developed countries [44]. Indeed, food availability is such that individuals have a wide choice of what to eat. On the other hand, the adaptation of consumer behavior to modern lifestyles has led to the demand for safer and longer-lasting food; this, in turn, has driven industry to increase the use of additives and develop new preservation technologies. Cooking has become a secondary concern, as the consumption of and demand for pre-cooked and ready-to-eat products has exponentially increased. These products must be tasty as well as easy to prepare and store; in this context, the addition of fats, sugar, and salt is imperative in meeting these requirements [45]. In addition, competitive markets force producers to use cheap ingredients in processed foods, and these are hardly ever the healthiest ones [46]. All these changes underlie the food industry’s transition from the natural products consumed by our ancestors to the processed products currently available, which tend to be high in artificial and added ingredients such as preservatives, colorants, fats, sugar, and salt (Figure 2) [44,47,48].

### 2.4. What the Numbers Say

**Figure 3:**
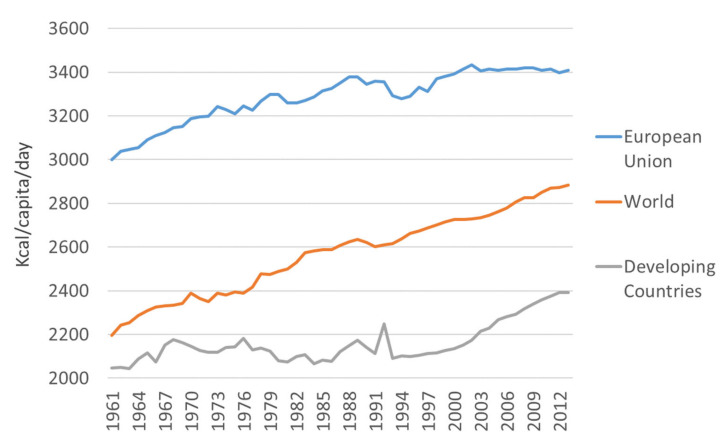
Increase in calorie intake in different regions over the last 50 years.

Global industrialization has facilitated these changes in diet and, notably, similar changes are observed in many countries despite differences in culture, lifestyle, and culinary traditions. According to the annual food balance sheets published by the Food and Agriculture Organization (FAOSTAT database; http://www.fao.org/faostat/en/#home), calorie intake has been increasing over recent decades. From the 1960s to the present (last data from 2013), the world’s average energy intake has increased by nearly 500 kcal per capita per day (Figure 3). The origin of this calorie increase is slightly different in developed and developing countries. While the consumption of meat, sugars, and vegetable oils has increased in developing countries, developed countries have seen rises in meat and fat intake [44]. The Food and Agriculture Organization data show that the increase in energy intake in European Union countries is approximately twice that observed in developing countries (Table 1).

The dietary pattern involving a high intake of saturated fats and sucrose and a low intake of fiber is commonly known as a “Western diet”. Diet is one of the strongest modulators of chronic inflammation and Western diets represent a growing health risk, contributing to higher rates of metabolic diseases and inflammation [31,47,48].

## 3. Human Gut Microbiota Evolution

The human microbiota has been structured by its biological interaction with its host, and the resultant ecosystem is the consequence of thousands of years of evolution [49]. Furthermore, microbes have impressive abilities to spread, interact, and adapt to the environment; hence, microbial communities should not be considered in isolation, but rather as part of an interacting community [50]. The knowledge in this relatively new field is still quite limited. In fact, it is difficult to know the extent to which the human microbiome has been shaped by the selective pressure of modern diet, hygiene, antibiotic exposure, built environment, and lifestyle [51]. Despite these limitations, the following paragraphs attempt to outline the current knowledge on gut microbiota composition and its evolution.

### 3.1. Defining “Healthy Microbiota”

In general, it is accepted that only four bacterial phyla dominate the human microbiota (Firmicutes, Proteobacteria, Actinobacteria, and Bacteroidetes), while others (Chlamydiae, Cyanobacteria, Deferribacteres, Deinococcus–Thermus, Fusobacteria, Spirochaetes, or Verrucomicrobia) may be found at lower abundances [52,53,54,55]. Strict anaerobes, mainly represented by members of the phyla Bacteroidetes and Firmicutes, dominate the gut, outnumbering aerobe microorganisms by 100- to 1000-fold [56,57,58,59,60]. Facultative anaerobes account for less than 1% of the microbiota and are mainly represented by the family *Enterobacteriaceae* and the genera *Enterococcus* and *Lactobacillus* [19].

Despite research efforts, there is no consensus on a detailed description of a “normal” or “healthy” microbiota. The enormous complexity and inter-individual variability in the microbiota make this goal very hard to achieve with current tools. Microbial richness (number of species) and diversity (variety and relative abundance of the species in a niche) are global parameters associated with health. Stability has also been considered as a key feature of a healthy microbiota, and this is related to the concepts of resistance (ability of a community to resist change in the context of ecological stress) and resilience (its ability to return to an equilibrium state following a stress-related perturbation).

Nevertheless, the idea that there is an ideal composition of the microbiota seems too simplistic. In fact, it minimizes the importance of microbiota–host interactions, individual genomic differences, and variations in susceptibility to disease, all of which probably play a determining role in shaping the microbiota. An alternative concept consists of characterizing the collection of genes and metabolic pathways provided by the microbiome rather than just the microbiota composition. This approach is probably more appropriate, but also requires a greater in-depth knowledge of the human microbiome [59].

In any case, it should be a priority to reach a consensus on the definition of a healthy microbiota in order to clarify the goal of strategies for microbiota modulation.

### 3.2. Clustering Individuals According to Their Microbiota Composition

As a step towards defining the composition of a healthy gut microbiota, an interesting publication clustered the fecal microbiota of a healthy cohort into three so-called “enterotypes”. Each cluster was characterized by the presence of some highly abundant genera that defined the group and many less abundant genera. Enterotype 1 was enriched in the genus *Bacteroides* and enterotype 2 in *Prevotella*, while enterotype 3 was dominated by *Ruminococcus*. The dominant genera tended to be observed together with other minority ones (*Parabacteroides*, *Desulfovibrio,* and *Akkermansia,* respectively) that, despite their low abundance, performed specialized functions beneficial to the host and are important for defining the enterotype. Though each enterotype preferred certain routes for generating energy, which suggests a specialization to their ecological niches [54], the data available support the idea that the gut microbiota is characterized by a high functional redundancy. In fact, 25% to 43% of the enzymatic functions of the microbiota have been found to be shared, regardless of the enterotype to which the microbiota belonged [21,60].

Some years later, another publication associated these enterotypes with long-term diets. The *Bacteroides* enterotype was strongly associated with a variety of amino acids from animal proteins and saturated fats, and therefore with Western diets. In contrast, the *Prevotella* enterotype was closely associated with carbohydrates and simple sugars, indicating an association with typical diets of agrarian societies [25].

A recent publication uses a metagenomic approach to classify individuals according to the number of gut microbiota-encoding genes as “low gene count” (LGC) or “high gene count” (HGC), depending on whether their microbiota harbor fewer or more than 480,000 genes, respectively. This approach is based on the functionality of the microbiota and its relation with the microbiota composition. The difference in the mean number of encoding genes between groups is notably high, reaching some 40%, and is related to the microbial richness. Broadly, LGC individuals have a less rich microbiota, dominated by *Bacteroides*, *Parabacteroides*, *Ruminococcus*, *Campylobacter*, *Dialister*, *Porphyromonas*, *Staphylococcus, Anaerostipes* and most members of the phylum Bacteroidetes. In contrast, the phylum Firmicutes and the genera *Faecalibacterium*, *Bifidobacterium*, *Lactobacillus*, *Butyrivibrio*, *Alistipes*, *Akkermansia*, *Coprococcus,* and *Methanobrevibacter* are associated with HGC individuals [61,62].

### 3.3. Ancient Microbiota

Regarding the changes in our gut ecosystem over the course of human evolution, some studies suggest that these are both pronounced and worrying. The study of the ancient microbiota is not easy due to the low number of available ancestral biological samples. The gut microbiota of ancestral specimens was evaluated in mummies, revealing the predominance of species of the genera *Clostridium* and *Bacteroides* in the larger intestine [63,64,65]. These studies provided valuable information, however, in addition to the small sampling size, the storage conditions and the possible post-mortem alterations in the bacterial communities should be considered in the interpretation of the results. In this context, current hunter-gatherer populations are also being studied. Research with uncontacted Amerindians who continue to live a seminomadic hunter-gatherer lifestyle revealed that their fecal microbiota is the most diverse ever reported in humans, and the proportion of shared microbiota between them is also much higher than in other human populations [32]. This high microbial biodiversity was also observed in studies carried out in other hunter-gatherer populations, such as the Matses from the Peruvian Amazon [66], the Hadza from Tanzania [33], or indigenous ethnic groups from the Central African Republic [36]. The Ameridians’ microbiota seems to be characterized by a high abundance of the phyla Verrucomicrobia and Mollicutes; the families *Aeromonadaceae*, *Oxalobacteraceae,* and *Methanomassiliicoccaceae;* and the genus *Prevotella*, while the abundance of the genus *Bacteroides* is lower [32]. The microbiota of the Matses is characterized by the abundance of the genera *Clostridium*, *Catenibacterium*, *Eubacterium*, *Lachnospira,* and *Treponema* [66]. The Hadza population presented a microbiota enriched in *Prevotella*, *Succinivibrio*, *Treponema,* and *Eubacterium* and impoverished in *Bacteroides*, *Blautia,* and *Dorea* genera [33]. The Central African Republic hunter-gatherer population’s microbiota is characterized by the predominance of *Prevotella* and *Treponema* [36]. Despite the differences, it is worth noting that the abundance of *Prevotella*, *Treponema,* and *Eubacterium* and the scarcity of *Bacteroides* in the microbiota of these populations may be common characteristics of the ancestral microbiota.

Metagenomic approaches allow us to analyze the genetic composition and function of complex communities. The application of these tools to the ancient microbiota provide further evidence to support the view that it has a higher functional diversity, characterized by increased metabolic pathways involving amino acid metabolism; glycosyltransferases; and the biosynthesis of lipopolysaccharides, terpenoid-quinones, and vitamins [32]. These findings suggest that not only is microbial diversity being lost, but also some of the functionality of gut microbials. As a consequence, it is not surprising that there is growing interest in ancient microbiome research and recovery [22,51,67].

### 3.4. Western Diet Microbiota and Its Consequences

Evidence suggests that lifestyle changes, including poor diet, urbanization, scarce physical activity, built environment, wide-spread antibiotic exposure, and better hygiene, have impacted the composition of our microbiota and also the emergence of the so-called diseases of modern civilization. These changes are included in the concept of “Westernalization” and contribute to microbiota alteration and disease [68]. Even if all aspects of Western lifestyle should be considered in this process, diet is accepted as one of the most potent ones shaping microbial communities [68,69,70].

There is a tendency to lose the overall diversity of the gut microbiota in people following Western diets. The gut microbiota composition has also undergone specific changes, characterized by an increase in the abundance of the phylum Firmicutes and the family *Enterobacteriaceae* and a decrease in the phylum Actinobacteria and the genus *Prevotella*. The presence of some bacterial species associated with anti-inflammatory conditions and the capacity to produce beneficial metabolites is diminishing in our guts. These species include *Akkermansia muciniphila*, *Faecalibacterium prausnitzii, Roseburia spp*., *Eubacterium hallii, Clostridium clusters XIVa* and *IV,* and *Ruminococcus*, among others. Indeed, some research has revealed the extinction of several bacterial groups from the guts of people following Western diets. It is difficult to assess the significance of that loss, but we are probably witnessing just the beginning of its consequences [12,31,47,71].

Furthermore, the gut microbiome’s circadian rhythm is influenced by factors such as light–dark cycles, sunlight exposure, sleep, and dietary patterns; some of them are common stressors of the modern lifestyle [72]. The consumption of food in an undisturbed daily rhythm coincides with the light phase of the light–dark cycle and the activity phase of the day, which has consequences on the regulation of the hosts’ intestinal cell transcription, the rhythms of the circulating metabolites, and the gut microbiota composition and function [72,73]. Some studies evidenced exacerbated effects on the gut microbiota of people with the circadian disruption of high sugar and fat diets; these effects were characterized by a drastic reduction in bacterial diversity and the Firmicutes/Bacteroidetes ratio [74,75].

The aforementioned changes in microbiota composition have been associated with a greater tendency to develop inflammation and, in turn, with a higher incidence of obesity; diabetes; allergies; cardiovascular disease; and metabolic, gut, and neurological disorders. Microbiota dysbiosis in these diseases may be involved in the alteration of certain specific microbial groups; nevertheless, in most cases the overall loss of microbial biodiversity is an important factor defining the dysbiosis [76]. The misalignment of the rhythms that control our energy metabolism also increases the risks of suffering diseases such as metabolic syndrome, including type 2 diabetes mellitus and obesity [77,78]. The growing incidence of these diseases in contemporary, industrialized populations over recent decades is believed to be associated, among other factors, with a lack of adaptation of our metabolism to the rapid dietary and lifestyle changes that have occurred over the course of human evolution [22,32,51,79,80,81].

It is likely that several different factors are contributing to the changes in microbiota composition and the increased prevalence of associated diseases. In any case, the impact of these changes on human health underlines the urgent need to find effective tools to halt this trend.

## 4. Modulation of Human Gut Microbiota with Diet

While it has already been described that geographical localization, culture, and genetic background all affect the microbiota composition, some authors consider that diet is responsible for more than 50% of the variability in the microbiota [82,83]. Even though it is difficult to determine this value accurately, there is evidence that dietary interventions, with significant changes in content, are able to exert modulatory effects on microbiota composition that may be seen within 1–4 days and are strong enough to shift the enterotype [84,85]. Nevertheless, dietary modulatory effects are diluted over time when the diet is discontinued, and there is a tendency to return to the original state [82].

The capacity of microbiota to recover its original status has also been observed after a course of antibiotics. Several studies have evidenced that some weeks after the use of antibiotics (one of the treatments that most dramatically alter the microbiota), the microbiota has nearly completely returned to its original composition, though this recovery is treatment- and age-dependent [86,87,88]. The frequent use of antibiotics or the requirement for prolonged treatments has a more marked effect on the microbiota composition [89,90]. Similarly, the modulatory effect of probiotics (live microorganisms which, when administered in adequate amounts, confer a health benefit to the host [70]; dead microbes, microbial products, or microbial components do not come under the probiotic classification [91]) is believed to disappear progressively together with the loss of the beneficial strains.

All this evidence supports the idea that treatments to modulate gut microbiota must be maintained over time. In line with this, the use of diet as a modulatory tool could be ideal whenever diet is considered as a long-lasting change in everyday habits.

### 4.1. Strategies for Modulating the Microbiota: Prebiotics

Possibly the most widely explored strategy for modulating the microbiota is the use of prebiotics. Prebiotics are defined as “substrate that is selectively utilized by host microorganisms conferring a health benefit” [92]—that is, nutrients resistant to gastric acid secretion and digestive enzymes that once in the gut stimulate the growth of beneficial microbes or their activity. Certain dietary components such as inulin, fructooligosaccharides (FOS), galactooligosaccharides (GOS), and resistant starch (RS) have been studied as prebiotics, and their efficacy is commonly indirectly measured by the production of short chain fatty acids (SCFAs) or the decrease in intestinal pH [76,93]. Inulin is a fructan carbohydrate that may vary its polymerization degree and whose fructose chains ranges from 2 to 60 monomers [94]. Inulin stimulates the growth of lactobacilli and bifidobacteria; besides this, an increase in *F. prausnitzii* and *A. muciniphila* populations in the gut has been described and seems to produce early satiety by modulating the gut endocrine function. Nevertheless, it is still difficult to determine the mechanisms underlying these effects [93].

The FOS are oligosaccharides of glucose and fructose that differ from inulin in their polymerization degree that is under 10; whereas, GOS are oligosaccharides of glucose and galactose with a polymerization degree of 2 to 8. As typical prebiotics, FOS and GOS have been used to stimulate the growth of the beneficial bacteria, bifidobacteria and lactobacilli. The administration of FOS in a culture-dependent study resulted in an increase in *Bifidobacterium* and *F. prausnitzii*, while culture-independent studies based on high-throughput sequencing have revealed changes in more than 100 bacterial taxa. The most marked changes in abundance were an increase in *Bifidobacterium;* reductions in the genera *Phascolarctobacterium*, *Enterobacter*, *Turicibacter*, *Coprococcus,* and *Salmonella*; an overall increase in Bacteroidetes; and a decrease in the phylum Firmicutes [95]. Other genera that could be increased by FOS administration are *Lactobacillus* and the butyrate producers *Faecalibacterium*, *Ruminococcus,* and *Oscillospira* [96]. On the other hand, GOS administration resulted in increases in *Bifidobacterium* levels and decreases in the levels of *Ruminococcus*, *Dehalobacterium*, *Synergistes,* and *Holdemania* [95]. The effect of GOS on the gut microbiota could also improve the butyrate production and the presence of butyrate producers, such as *Eubacterium rectale* [97].

RS is defined as the total amount of starch and the products of starch degradation that resists digestion and has been shown to be composed of a linear molecule of α-1, 4-D-glucan, derived from the retrograded amylose fraction. Various classifications have been proposed for RS based on four or five types, and the content of RS in foods is influenced by the physical form of the food, the size and composition of the starch granules (amylose–amylopectin ratio), and the food processing methods and conditions [98,99]. Notably, it has been found that an increment in RS in the diet is associated with colonization by higher levels of the phylum Bacteroidetes and the genera *Bifidobacterium*, *Akkermansia,* and *Allobactum* [98].

### 4.2. Strategies for Modulating the Microbiota: Probiotics

The field of probiotics—which, as stated above, are live microorganisms which when administered in adequate amounts confer a health benefit to the host [70,91]—has notably grown in recent years. The microorganisms commonly used as probiotics are the yeast *Saccharomyces cerevisiae* and members of the bacterial genera *Lactobacillus* and *Bifidobacterium*, though some formulations may also include some *Streptococcus*, *Enterococcus*, *Pediococcus*, *Propionibacterium*, *Bacillus,* or *Escherichia* strains. Most *Lactobacillus* and *Bifidobacterium* species have been assigned “Generally Recognized As Safe” status by the US Food and Drug Administration and “Qualified presumption of safety” status by the European Food Safety Authority, facilitating their preferential use as probiotics. On the other hand, their long history of use as probiotics means that there is a substantial body of evidence for a wide range of beneficial properties [100], though we must recall that probiotic properties are strain-specific—that is, they are not a characteristic of a species [76].

Nevertheless, it can be expected that, in the near future, other species will be used as probiotics—ones that are more commonly found in the human gut and play important functions in mitigating intestinal inflammation, inducing immune regulation, or enhancing the intestinal barrier function. These are likely to include species with anti-inflammatory properties (*A. muciniphila*, *F. prausnitzii*) and butyrate-producing bacteria [91,101].

Currently, probiotics are used in a wide range of contexts, normally being indicated for healthy people in a special situation (e.g., during infancy, pregnancy, breastfeeding, and old age) and for preventing or treating several specific health problems. As a consequence, the assessment of the safety of probiotics must pay attention not only to the selected strain, manner and frequency of administration, and dose and treatment duration but also to the potential vulnerability of the consumer and the physiological function that the strain may play in the host [102].

Despite the heterogeneity of clinical studies making it difficult to determine the most suitable strains and therapeutic guidelines, there is clear evidence of probiotic effectiveness in the prevention or treatment of diseases such as necrotizing enterocolitis, antibiotic-associated diarrhea, colitis, or acute gastroenteritis [103,104]. Nonetheless, to date there is a lack of evidence of the effectiveness of prebiotics or probiotics in achieving long-term changes in the microbiota.

### 4.3. Strategies for Modulating the Microbiota: Controlling the Gut Environment

The concept of gut environment modulation considers not only the composition of the microbiota but also its function and interaction with the host. Nowadays, it is known that fat-rich diets enhance bile secretion to facilitate lipid digestion. Bile acids have antimicrobial properties; their detergent effect produces damage to bacterial membranes and exerts a strong selective pressure on microbiota composition. Studies carried out in rats reveal the strong resistance of the phylum Firmicutes to bile acids, in particular, the classes *Clostridia* and *Erysipelotrichia* and the family *Enterobacteriaceae* [105]. These findings are in agreement with the predominant presence of Firmicutes in people following Western diets [79].

On the other hand, intestinal pH may oscillate between 5 and 7 under normal physiological conditions, depending on the fermentation products present (such as SCFAs) and the metabolite absorption by host epithelial cells and their level of bicarbonate secretion. Intestinal pH affects not only the microbiota composition but also its metabolism. Some Firmicutes species, especially those belonging to *Clostridium cluster XIVa,* are tolerant of a low pH; however, many Bacteroidetes and Actinobacteria members are more sensitive to pH changes [12,106].

The Mediterranean diet is associated with a higher production of SCFAs in the gut [107,108]. These substances play important roles maintaining the integrity of the large bowel and small intestinal barrier, providing energy to epithelial cells and reducing inflammation [108] and support higher microbial richness in the gut [107,109].

The modulation of the gut microbiota through the induction of environmental changes has been less explored; but the gut environment remains closely related to dietary pattern and therefore should not be disregarded in future dietary interventions.

### 4.4. Challenges in Microbiota Modulation: Interindividual Variability

The complexity of gut microbiota modulation lies in interpersonal variability not only in the microbiota composition and functioning but also in differences in lifestyle and genetic predisposition to disease. It has been described that changes in fiber consumption lead to various changes in the gut microbiota. In general, the abundance of the family *Lachnospiraceae* increases with the incorporation of insoluble fiber or wheat bran to the diet, while enrichment in RS causes an increase in *Ruminococcaceae*, but changes are individual specific [62]. Along the same lines, a recent study revealed that changes in oral glucose tolerance and the microbiota induced by a given prebiotic intervention vary between individuals. Notably, a close positive correlation was found between glucose tolerance and the presence of some butyrate-producing bacteria [95].

These data indicate the enormous importance of knowledge of the microbiota composition to predict the effects of modulatory treatments. The available data suggest that parameters such as age, diet, and lifestyle, body mass composition, and the presence of specific diseases could help to define some key features of the microbiota composition, but treatments to modulate the microbiota are still based on estimates and it is likely that some effects are being masked.

### 4.5. Challenges in Microbiota Modulation: Microbial Metabolic Redundancy

To estimate the effects of a modulating treatment, in addition to microbiota composition there is a need to consider metabolic redundancy. The use of next-generation sequencing and metagenomics is greatly helping to advance our understanding of this factor. In this context, recent publications describe the metabolic capacity of some bacterial groups. It seems that members of the genus *Bacteroides* have a wide metabolic arsenal allowing them to utilize different polysaccharides, whereas Firmicutes members have less metabolic diversity for polysaccharide degradation and greater nutritional specialization [62].

Metabolic analyses of LGC and HGC individuals suggest a great capacity to handle oxidative stress but also a higher production of detrimental metabolites and a predisposition to inflammation in LGC individuals. On the other hand, HGC individuals’ microbiota has a greater capacity to produce organic acids, including SCFAs. Further, it seems that there is a higher incidence of obesity and metabolic syndrome in LGC individuals and dietary interventions for weight loss increase microbiota diversity and the overall gene counts [61,62,110].

In accordance with this, the analysis of the metabolic specialization of the enterotypes indicated that individuals with the *Bacteroides* enterotype are better able to digest lipids, proteins and carbohydrates of animal origin, while the *Prevotella* enterotype showed a plant fiber hydrolysis specialization. In contrast, the *Ruminococcus* enterotype has no such marked specialization, but has been linked to a higher microbiota diversity and a lower inflammatory status in the host [84].

The aforementioned results suggest a healthy microbiota should be closer to that of HGC individuals and the *Ruminococcus* enterotype. Nonetheless, no consensus has yet been reached on the definition of a healthy microbiota. In fact, all enterotypes have been associated with some types of disease and it is believed that each enterotype has a different susceptibility to given illnesses. In any case, a greater knowledge of individuals’ microbiota could be a good tool to guide treatment decisions and estimate patient response.

An alternative more affordable approach to exploring the microbiome function without resorting to metagenomics is the measurement of SCFA production. These metabolites are considered important for their influence on intestinal homeostasis, pH, and preventing the growth of potentially pathogenic bacteria. Moreover, SCFAs have anti-inflammatory and anti-apoptotic properties, contribute to the gut barrier integrity, and are key elements of the gut–brain axis. These fatty acids seem to be necessary for the proper maturation and functioning of microglia, also known as brain macrophages, that participate in brain physiology and homeostasis have phagocytic capacity and also participate in the integrity of the blood–brain barrier [53,111,112,113].

Microbiota-derived metabolites such as SCFAs depend directly on diet; mainly on its content of non-digestible carbohydrates, lipids, and proteins and the metabolic pathways available in the microbiome [12,62]. Some common microorganisms of the gut microbiota and the SCFAs that they produce are listed in Table 2. Individual dietary choices influence not only gut microbiota composition but its function, but we still lack an understanding of how the microbiome interacts with nutritional and host-genomic axes to confer predisposition to disease.

## 5. Conclusions

Diet and lifestyle habits have undergone dramatic changes since the origin of humanity. The first hominid’s diet was based on the consumption of raw vegetables collected from the wild and a low intake of protein of animal origin. After thousands of years of evolution, the modern diet, influenced by globalization and consumerism, is characterized by an excessive lipid and energy intake and the introduction of processed and refined foods that are rich in lipids, sugars, salt, and preservatives. These foods have facilitated the accelerated pace of today’s life, but it is likely that their negative consequences for human health are just beginning to be noticed.

Despite the relative youth of microbiome research, microbiota dysbiosis has already been associated with diverse health problems. Numerous international projects are focusing their research efforts on the study of human microbiome in different contexts, including the Human Microbiome Project (HMP: https://www.hmpdacc.org/hmp/), the International Cancer Microbiome Consortium (ICMC: https://www.icmconsortium.org/), the International Multiple Sclerosis Microbiome Study (iMSMS: http://imsms.org/home/), and the Inflammatory Arthritis Microbiome Consortium (IAMC); more are likely to be launched in the near future. Diet has been identified as one of the factors with the greatest influence on microbiota acquisition (in newborns) and modulation. Therefore, detailed studies of the complex interactions that occur between diet and microbiota are necessary to effectively direct desirable changes in human microbiota or alter its abnormal composition in disease. So far, making simple predictions of dietary effects remains extremely difficult.

Microorganisms, especially prokaryotes, are characterized by their amazing capacity to adapt to the environment, as evidenced by their ability to remain stable in an “altered microbiota”. Human beings seem to be much less efficient at environmental adaptation. Certainly, the growing rate of diseases related to compromised immune or nervous system function and alterations in metabolism may be a response to a lack of adaptation to microbiota dysbiosis.

Most of the strategies designed to modulate the microbiota are based on one-off treatments that could be effective during their administration, but do not seem to produce stable changes in the microbiota, maybe with the exception of a fecal transplant (although there is still challenges with this practice [114]). In this context, diet will probably be the most powerful tool for microbiota modulation, but to achieve that a better understanding of diet–host–microbiota interactions is necessary. Learning how to use diet to generate a healthy microbiota must be a priority for society, and arguably represents one of the key steps to achieving real preventive and personalized medicine.

## Figures and Tables

**Table 1:** Energy intakes in the last five decades (kcal/capita/day).

Area	Item	1961–1970	1971–1980	1981–1990	1991–2000	2001–2010	Variation from the 60s to 2010
**European Union**	**TOTAL**	3094.9 (59.4)	3241.3 (37)	3316.9 (46.2)	3336.5 (41)	3415.1 (8)	**320.2**
	**Plant-based products**	2223.7 (23.7)	2257.2 (14.5)	2272.6 (35.7)	2321.6 (32.8)	2407.5 (11.6)	**138.8**
	**Animal products**	871.2 (37.5)	984.2 (31.2)	1044.4 (13)	1014.8 (17.7)	1007.7 (11.2)	**136.5**
	**Sugar and sweeteners**	339.7 (18.9)	378.5 (7.8)	365 (6.1)	364.3 (2.8)	373.6 (9.3)	**33.9**
	**Vegetable oils**	273 (18.4)	322.9 (11.6)	373.6 (22)	431.1 (13.2)	456.3 (15.6)	**183.3**
	**Meat**	282.3 (20.5)	355.4 (19.6)	388.2 (12.1)	393.8 (7.7)	385.3 (4.8)	**103**
	**Animal fats**	235.4 (6.7)	242.6 (3.5)	242.3 (6.5)	214.6 (6.9)	196.9 (6.4)	**−38.5**
	**Milk**	270.6 (7.3)	290.5 (7.3)	312.2 (6.6)	305.7 (5.7)	317.4 (2.3)	**46.8**
**Developing Countries**	Total	2105.6 (51.3)	2130.7 (25.8)	2109.4 (35.7)	2125 (45.2)	2262.9 (69.4)	**157.3**
	**Plant-based products**	1914.3 (44.2)	1933.8 (28.2)	1918.9 (39.9)	1886.1 (31.7)	2011.8 (54.9)	**97.5**
	**Animal products**	191.2 (8.3)	196.8 (5.7)	190.4 (11)	239 (26)	251.1 (15.1)	**59.9**
	**Sugar and sweeteners**	71.2 (9.9)	78.9 (4.9)	90.6 (3.3)	103.2 (7.8)	107.8 (5.7)	**36.6**
	**Vegetable oils**	52.7 (4.1)	66.2 (5.7)	86.9 (11)	114.8 (5.9)	137.5 (12.4)	**84.8**
	**Meat**	79.2 (3.6)	81.1 (3.5)	84.1 (3.4)	100.1 (10.3)	98.5 (5.3)	**19.3**
	**Animal fats**	27.8 (2.5)	29.5 (1.2)	27.2 (2.5)	28 (5)	25.6 (1.7)	**−2.2**
	**Milk**	67.4 (1.9)	66.4 (1.4)	59.9 (5.2)	91.7 (12.8)	106 (6.4)	**38.6**

Values expressed as mean and standard deviation.

**Table 2:** Microorganisms that produce short chain fatty acids and other fermentation products.

Microorganism		Short Chain Fatty Acids and Other Fermentation Products							
Phyla	Species	Acetate	Propionate	Butyrate	Ethanol	Formate	Lactate	Butanol	Succinate
**Firmicutes**	***Eubacterium rectale***			X		X	X		
	***Roseburia inulinivorans***		X	X		X	X		
	***Eubacterium hallii***			X		X		X	
	***Anaerostipes hadrus***			X					
	***Coprococcus catus***		X	X					
	***Ruminococcus obeum***	X					X		
	***Blautia wexlerae***	X							X
	***Faecalibacterium prausnitzii***			X		X	X		
	***Ruminococcus bromii***	X			X				
**Bacteroidetes**	***Bacteroides thetaiotaomicron***	X	X						X
	***Bacteroides vulgatus***	X	X						X
**Actinobacteria**	***Bifidobacterium adolescentis***	X				X	X		
	***Collinsella aerofaciens***	X					X		

Adapted from “Links between diet, gut microbiota composition and gut metabolism” by Flint HJ et al. [60].
